# How students' writing motivation, teachers' personal and professional attributes, and writing instruction impact student writing achievement: a two-level hierarchical linear modeling study

**DOI:** 10.3389/fpsyg.2023.1213929

**Published:** 2023-07-20

**Authors:** Heqiao Wang, Gary A. Troia

**Affiliations:** Department of Counseling, Educational Psychology and Special Education, Michigan State University, East Lansing, MI, United States

**Keywords:** writing achievement, teacher efficacy beliefs, instructional actions, writing motivation, hierarchical linear modeling

## Abstract

Student motivation to write is a pivotal factor influencing their writing achievement. However, individual motivation to write is not independent of the learning environment. It also is crucial for teachers to develop their own efficacy, knowledge, and ability in writing and writing instruction to help them utilize effective instructional methods that stimulate students' motivation to write and further promote their writing achievement. Given these considerations, we utilized a two-level hierarchical linear model to examine the relationships among student motivation, teacher personal and professional traits, teacher writing instruction, and writing achievement at student and teacher levels. Our analysis of the dataset, which included 346 fourth and fifth graders nested within 41 classrooms, found that motivation had a positive predictive effect on writing ability at both student and teacher levels. Moreover, female students, fifth graders, and typically achieving students demonstrated higher writing achievement than their counterparts. While there were no significant effects of teacher efficacy, knowledge, ability, or professional development on student writing achievement, we observed that higher frequency of classroom management practices during writing instruction had a significant negative effect on student writing achievement. Our full model revealed that the relationship between student motivation and achievement was negatively moderated by teachers' increased use of instructional practices related to process features and using writing instruction materials, but positively moderated by increased use of varied teaching tactics. Overall, our findings emphasize the importance of contextual factors in understanding the complexity of student writing achievement and draw attention to the need for effective instructional practices to support students' writing development.

## 1. Introduction

The development of proficient writing skill is widely recognized as an indispensable component of K-12 education in the United States, as it empowers individuals to attain their academic, occupational, and personal aspirations (Graham, 2019; Sato and Thompson, 2020). However, the majority of young learners do not achieve mastery in the requisite writing behaviors and skills aligned with their grade-level expectations (Deane, 2011). This concerning trend is corroborated by the findings of the Nation Center for Education Statistics (NCES), which measures writing performance using the National Assessment of Educational Progress. The 2011 Nation's Report Card revealed that only 27% of twelfth-grade students demonstrated proficiency in writing, indicating a pervasive deficiency across the nation in constructing written responses that effectively accomplish the communicative purpose of writing, with proficient writing characterized by well-organized and coherent text with appropriate transitions and diverse sentence structure (NCES, 2011; Crossley and McNamara, 2016). In addition, half of learners encounter difficulties in even the most rudimentary aspects of writing, such as using detailed and factual descriptions, appropriate lexical choice, and varied sentence structures (NCES, 2011). The unprecedented decline in average scores across other core academic subjects (i.e., mathematics and reading) during the COVID-19 pandemic years, as reported in 2022 by the NCES, has further exacerbated concerns regarding writing deficiencies in the student population.

Examining the multitude of factors that influence writing performance represents a complex endeavor. Of these factors, student-level factors have garnered considerable research attention, given their direct and substantial influence on writing achievement (e.g., Maxwell et al., 2017; Coker et al., 2018). The existing writing models provide theoretical frameworks for understanding how the acquisition of writing skills and the production of written text can be influenced by individual factors. One such notable model is Hayes' (1996) cognitive model of writing, which underscores the central role of motivation and its enduring impact on student writing performance throughout the entire writing process. The model posits that motivation can facilitate both short-term responses to immediate writing goals and a long-term predisposition to engage in writing activities, even when they present challenging demands. Additionally, the model incorporates other individual factors, such as writing knowledge, working memory, and the ability to transcribe and translate ideas into conventional linguistic units, to account for the complexity of writing performance. Empirically, research has identified individual characteristics, such as motivational beliefs (e.g., Graham et al., 2018), writing knowledge (e.g., Saddler and Graham, 2007), working memory (e.g., Cordeiro et al., 2020), and writing-related behaviors and strategies (e.g., Graham et al., 2017b; Wijekumar et al., 2019), as significant contributors to writing achievement on the individual level.

Meanwhile, individual differences in writing-related factors are contingent upon the environment and are amenable to change through teachers' personal and professional qualities, as well as their instructional practices. Extensive research shows that teachers' beliefs in their ability to write and teach writing effectively (e.g., Tschannen-Moran and Barr, 2004; Corkett et al., 2011), writing knowledge and abilities (e.g., Huang and Shimizu, 2016), and participation in professional development programs (e.g., Roberts, 2002; Fearn and Farnan, 2007), have a positive and lasting impact on their students' writing performance and development. Moreover, establishing a supportive and inclusive learning environment by adopting effective writing instructional practices (e.g., Lam and Law, 2007; Graham and Harris, 2013; De Smedt and Van Keer, 2014), incorporating cultural and linguistic diversity elements when designing writing curricula and assessments (e.g., Datnow et al., 2003; Shapiro et al., 2016), and organizing school-wide celebratory events (e.g., Bradshaw et al., 2009) can also promote writing success. These findings also resonate with Graham's (2018) writers-within-community perspective, which emphasizes the significance of contextual factors and writing communities in shaping the meaning, motivation, and effectiveness of writing. Effective writing instruction should not only align with individual goals but also consider the intended audience, norms, and conventions of the genre to enhance the quality of writing. To accomplish this, teachers are expected to possess pedagogical knowledge and attitudes for teaching quality writing and a deep understanding of the social policy forces that influence writing instruction (Troia et al., 2011; Harris and Graham, 2016).

Despite a substantial body of research exploring the effects of various factors on student writing achievement, the majority of studies have investigated the associations between writing achievement and influential factors at the student and teacher level independently, without considering their interactional effects (e.g., Graham et al., 2017a; Bresina and McMaster, 2020; Wright et al., 2021). To address the complex nature and multilayered structure underlying writing achievement, it is essential for research to examine the nested relationships and consider the interplay of variables at higher levels through adopting multilevel analyses to mitigate aggregation bias and heterogeneity of regression (Anderson, 2012). Although some studies have investigated writing achievement from an integrated perspective by considering multilevel effects (e.g., Olinghouse, 2008; Mo and Troia, 2017), there is still much to explore regarding how these cross-level effects contribute to the effectiveness of writing instruction and ultimately lead to improved student writing achievement and what types of writing instructional actions can impact students' writing performance when considering their varying levels of writing motivation.

## 2. Student-level predictors of writing achievement

### 2.1. Writing motivation

Writing motivation has been a well-established area of research within the educational field, with recent conceptualizations highlighting the critical motivational and affective forces shaping students' perceived gains and losses in writing performance (e.g., Troia et al., 2013). Empirical evidence consistently suggests that motivated students demonstrate positive and strategic behaviors toward writing (e.g., Conroy et al., 2009; Wijekumar et al., 2019), expend extra effort on writing assignments (e.g., Hidi and Boscolo, 2006; Troia et al., 2012), persist in undertaking challenging writing tasks (e.g., Schrodt et al., 2019), actively seek feedback and guidance from teachers and peers (e.g., Williams and Takaku, 2011), collaborate with others to share writing ideas (e.g., Turner and Paris, 1995; Graham et al., 2017b), self-regulate their learning to write (Zimmerman, 1990), and evaluate their drafts periodically (e.g., Boscolo and Hidi, 2006). These behaviors enable students to complete writing tasks successfully, resulting in longer and better texts and further reinforcing their enthusiasm for writing (e.g., Graham et al., 2018).

Research has provided compelling evidence of the significant and positive associations between writing motivation and outcomes. For instance, a meta-analysis conducted by Camacho et al. (2021) revealed that multiple motivational constructs, such as self-efficacy and attitudes toward writing, were moderately associated with writing performance. Conversely, the positive impact of performance on motivational levels has also been observed, as students who experience success in writing tasks tend to exhibit higher levels of motivation. A recent systematic review by Alves-Wold et al. (2023) investigated self-reported writing motivation, with a specific focus on K-5 students. The review found that motivational levels varied depending on students' ability level and that students' self-efficacy beliefs were positively related to their actual writing performance, with changes in performance affecting motivation levels. Additionally, the review examined the construct validity of student self-reported motivational scales and highlighted the importance of designing motivational measures that align with their intended purpose and design features.

### 2.2. Individual demographic characteristics

The impact of demographic factors such as gender, grade, and learning ability on student writing achievement has been extensively analyzed in the literature on writing motivation and achievement. Research has yielded a mixture of findings regarding gender differences in writing motivation. Girls tend to report higher levels of achievement-oriented goals and self-efficacy beliefs than boys, as they often attribute their successes to effort and hard work (Pajares et al., 2000). However, girls possess lower self-esteem than boys, and their expectations for success may be undermined as writing tasks becomes increasingly difficult (Hidi et al., 2002). Boys, on the other hand, tend to rate their confidence higher than girls, potentially due to their more positive beliefs about their own writing ability (Pajares and Johnson, 1996). There are a few studies that demonstrated no statistically significant differences between male and female students in certain motivational constructs, such as in self-efficacy beliefs. For example, other gender-related factors, such as gender orientation (i.e., stereotypical beliefs about gender and task performance that students usually hold; Pajares and Valiante, 2001), may confound the effects of gender on writing motivation and achievement. Hence, gender can be regarded as a proxy variable that is associated with motivational beliefs and can explain writing achievement.

Numerous studies have examined the relationship between grade level and writing motivation, with varying results. Generally, lower grade students exhibit higher levels of self-efficacy beliefs compared to their counterparts in higher grades. For example, Shell et al. (1995) discovered that fourth graders reported significantly higher levels of self-efficacy, effort, and intelligence than 7th and 10th graders. In contrast, 7th graders showed little difference compared with 10th graders, except for self-efficacy beliefs where there was a slight decrease among 10th graders. This tendency is consistent with other studies demonstrating that writing motivation may decrease as early as Grade 3 and remain stable through middle and high school (Koster et al., 2015; James et al., 2017). This decline in motivation could be attributed to the increasing difficulty of writing tasks (Boscolo and Gelati, 2019) and the attainment of more accurate self-perception (Stipek, 1993) as students' progress through school. Empirical investigations (e.g., Pajares, 2003; Pajares et al., 2007a) have consistently indicated a weakening trend in writing motivation among students as they advance in their academic careers. However, some studies have sought to identify the nuances of writing motives. For instance, Rasteiro and Limpo's (2023) research revealed that middle school students demonstrated greater confidence in their use of the conventions of writing than higher-level cognitive skills such as ideation and self-regulation. Furthermore, they observed that middle school students were motivated to engage in writing activities by a combination of intrinsic (e.g., curiosity) and extrinsic (e.g., assignment grade) factors. It is also noteworthy that the relationship between a writers' abilities and their level of motivation may shift as they gain more experience and proficiency in writing (Pajares et al., 2007b).

In addition, a student's learning ability can play a vital role in determining their level of motivation and achievement in writing. Individuals with higher learning ability often possess more advanced cognitive and metacognitive skills that allow them to comprehend and analyze complex texts, generate and organize ideas, and employ effective writing strategies (Karlen and Compagnoni, 2017). These skills can boost their confidence and motivation to engage in writing activities. Conversely, students with lower learning ability may struggle with these skills, leading to frustration and reduced motivation to write. They may also encounter difficulties in mastering basic writing techniques such as spelling, grammar, and punctuation, which can further impede their writing progress and diminish their confidence and motivational beliefs (Troia et al., 2009; Roitsch et al., 2021). Brouwer's (2012) study found that students experiencing language learning impairment had diminished perceptions of their writing competence and their autonomous writing motivation. Although language learning ability did not necessarily have a direct association with student writing motivation, it could function as a moderator that influences the connection between motivation and writing quality. This is because language learning ability influences the proficiency with which students can articulate their thoughts in written form and can further decrease their motivation and writing outcomes if impaired.

## 3. Teacher-level predictors of writing achievement

### 3.1. Teacher efficacy beliefs

Although the body of research on teacher-level factors influencing students' writing performance is not as extensive as that on student-level factors, it is equally important to recognize their role in promoting students' writing proficiency, positive learning environment, and motivational beliefs (Graham et al., 2001). A teacher's self-efficacy beliefs is one of the most critical teacher-level factors that can lead to effective writing instruction. It can manifest in various aspects. Firstly, teachers with a strong sense of self-efficacy are more likely to adopt evidence-based teaching approaches that are multimodal and innovative (Posnanski, 2002) and demonstrate empathy and cater to the diverse needs of their students (Goroshit and Hen, 2016). Secondly, teachers with strong efficacy beliefs can enhance writing curriculum and assessment by dedicating more time to teaching grammar, mechanics, and content-level skills, such as developing ideas and text structures (Handtke and Bögeholz, 2019; Wyatt and Dikilitaş, 2021). Furthermore, they can enhance classroom management by implementing strategies to motivate students to write (Mojavezi and Tamiz, 2012), organizing in-class events and discussions on writing (Myhill et al., 2013), managing their classes efficiently to prevent disruptive behaviors (Poulou et al., 2019), and avoiding overly criticizing student errors (Shaukat and Iqbal, 2012). Collectively, these practices can help emphasize the importance of writing within the class, increase student engagement and enthusiasm, and achieve desired writing instruction outcomes.

A teacher's sense of efficacy is also influenced by contextual factors beyond their personal capabilities, such as professional development and teacher training programs (Posnanski, 2002), school resources and materials (Lee et al., 2011), and statewide assessment policies and high-stakes testing (Gonzalez et al., 2017). Troia and Graham's (2016) national survey found that teachers' beliefs and attitudes toward the Common Core State Standards for Writing and Language (CCSS-WL) and Common Core Aligned Assessments for Writing and Language (CCAA-WL) were associated with their sense of efficacy for teaching. Teachers who exhibited strong self-efficacy beliefs for teaching tended to hold favorable perceptions of the CCSS-WL and viewed them as feasible to implement with effort. This alignment with state standards was viewed as supportive of students in achieving satisfactory academic outcomes. The survey suggested that teachers who possess a positive self-perception of their efficacy as educators and are adequately prepared to teach writing are more likely to perceive state standards as a means to achieve improved student writing outcomes rather than a barrier hindering their ability to implement effective teaching practices.

Additional scholarly findings suggest that teachers' self-efficacy beliefs for their writing abilities and writing instruction skills are both important indicators of their effectiveness as writing educators. To become efficacious, it is crucial for teachers to develop a solid understanding of writing skills development and possess the capability to effectively implement writing instruction in their classrooms (Grossman et al., 2009). Teachers who lack confidence in their ability to lead student learning effectively may avoid emphasizing the importance of writing to their students and may not allocate sufficient time for writing instruction (Tschannen-Moran and Hoy, 2001), which can have negative effects on their students' writing skills and motivation. Consequently, it is essential for teachers to develop their own writing skills and have confidence in their capacity to teach writing to their students.

### 3.2. Teacher professional development and writing expertise

Efficient writing instruction necessitates competent teachers with a strong knowledge base, skills, and strategies in writing. Nevertheless, there is a scarcity of literature on teachers' writing knowledge, and one approach to evaluate their teaching writing knowledge is to examine their training programs (Lembke et al., 2021). According to a national report by Yoon et al. (2007), professional development can positively affect student achievement by first influencing teacher knowledge and skills, which subsequently serves as a mediator, leading to higher student achievement. The report also reveals a moderate-to-strong effect size of 0.53 on reading and writing performance, underlining that effective professional development training or workshops can significantly impact student achievement in these academic areas. Hence, it is essential to evaluate the extent to which teachers have received and internalized such trainings while assessing the impact of professional development efforts on student writing achievement.

Previous research has established that professional development programs that address both beliefs and practices enable teachers to shape their pedagogies and translate their beliefs into effective teaching behaviors (Doubet and Southall, 2017). In a randomized controlled trial conducted by Myhill et al. (2013), 32 teachers from different schools were assessed on their grammar knowledge and pedagogical content knowledge through an achievement test and interview. It was found that teachers with extensive knowledge of grammar were better equipped to enhance learning outcomes and assist their students in developing metalinguistic comprehension of written discourse. On the other hand, teachers with limited grammar knowledge may encounter challenges in handling grammatical discussions, especially when confronted with students' inquiries, and could potentially overlook opportunities to rectify misunderstandings related to grammar usage.

### 3.3. Writing instruction actions

The implementation of effective instructional practices is paramount to minimizing the discrepancies between anticipated and actual student achievement outcomes (Guskey, 1982). However, the quality and quantity of writing instruction provided to K-12 students often falls short (Cutler and Graham, 2008). Graham (2019) identified four major indicators of insufficient writing instruction, including inadequate time allocated for teaching writing, particularly for unfamiliar writing tasks, infrequent opportunities for students to engage in writing activities, limited utilization of evidence-based writing instruction, and insufficient access to digital tools to support students' writing needs. Addressing these shortcomings requires a concerted effort, including teachers' commitment to enhance their expertise and attitudes, as well as radical changes in curriculum standards and associated instructional materials within the educational system. Although mitigating these inadequacies can be daunting, analyzing the interconnections between student- and instructional-level variables may yield meaningful implications for educational practitioners seeking to facilitate student writing outcomes.

Numerous experimental research and synthesis studies (e.g., Graham and Perin, 2007; Graham et al., 2012) have demonstrated that writing instruction can enhance text quality and quantity, and also spark students' creativity and interest in writing tasks, as long as specific components are incorporated. One key component is the process-oriented approach to teaching writing, which involves explicit instruction of various practices such as planning and revising writing components, peer conferencing for providing and receiving feedback on writing, sharing writing ideas with classmates, monitoring writing progress, selecting one's writing topics, working at one's own pace, and using invented spellings (Pritchard and Honeycutt, 2006; Cutler and Graham, 2008). According to a meta-analysis study by Graham and Sandmel (2011), process-focused writing instruction produced a statistically significant but modest increase in the overall quality of writing, as evidenced by an average weighted effect size of 0.34. Despite some studies reporting low effect sizes for certain writing processes and activities (e.g., traditional grammar instruction), the process approach to writing instruction remains a valuable albeit moderately influential strategy for teaching writing to students in general education classrooms.

Effective writing instruction can also be achieved through the use of appropriate teaching materials. Ciullo and Reutebuch (2013) found that interventions using technology-based graphic organizers or concept maps had a relatively high effect size of 0.80 in improving writing outcomes. By providing students with a structured method for planning and organizing their ideas, graphic organizers can enhance both the quantity and quality of their text output. Similarly, digital writing environments offer an immersive and interactive experience for students, leading to an increase in students' motivation, quantity and quality of writing, use of the writing process, and writing skills (Yamaç et al., 2020). Word processors are one such tool with digital environments, and they have been shown to be effective in improving writing length, quality, development and organization of text, mechanical correctness, and motivation to write (Morphy and Graham, 2012). These programs allow for easy revision and produce legible characters while providing additional learning supports such as speech recognition and spellcheck. Incorporating rubric-based feedback has also been observed to lead to higher levels of self-efficacy for elementary-aged students in writing class (Hier and Mahony, 2018).

Effective instruction in writing is also evident in both its teaching content and methods. An essential component involves incorporating instructional practices that encompass transcription, grammar, vocabulary, text structures, and general global features. These practices are crucial for improving students' overall writing quality and productivity. Specifically, in the early and middle elementary grades, it is imperative to prioritize the teaching of these basic composing writing skills as they establish a solid foundation for advancing writing abilities (Graham et al., 2012). Kim et al. (2021) meta-analysis study provides evidence that focusing on the basic mechanics and conventions of writing has a moderate and positive effect (ES = 0.31) on writing outcomes for primary-grade students (Kindergarten to Grade 3). This effect is particularly pronounced among students with weak writing skills compared to those with typical writing skills. Pedagogically, teachers can model the writing process and exemplify the desired products through the utilization of various material supports, such as writing notebooks, graphic organizers, checklists, and rubrics. Moreover, teachers can foster student engagement through questioning, offering suggestions, and facilitating summarization activities. These approaches contribute to enhancing students' writing proficiency and their understanding of effective strategies (Troia et al., 2011). In a study conducted by Graham and Perin (2007), explicit teaching interventions, such as summarization, were found to have a significant positive effect on writing outcomes, with an effect size of 0.82.

Effective writing instruction is also reliant upon successful classroom management and organizational skills. Elementary school teachers who possess these skills are more likely to have actively engaged students in their classrooms (Clunies-Ross et al., 2008; Rimm-Kaufman et al., 2015), leading to increased participation, greater persistence, and fewer behavioral issues (Rimm-Kaufman et al., 2009). Additionally, classroom management methods that provide clarity and consistency in class regulations have been shown to enhance student interest and emotional engagement in writing (Kunter et al., 2007; Hochweber et al., 2014). By incorporating these strategies into writing instruction, teachers can optimize student learning and academic achievements.

## 4. Interplay between student- and teacher-level predictors

Several studies have investigated the variability of students' writing achievement at different levels, including the student, class, school, and broader state levels. Most of these investigations have utilized multilevel modeling to account for the variance within the nested structure of the educational data, allowing them to examine the effects of various factors and their interactions that contribute to explaining achievement disparities between classes. For example, Olinghouse (2008) investigated the impact of student- and instructional-level factors on the variability of narrative writing fluency and quality. The study revealed that students with low word reading ability could benefit from intensive spelling and grammar instruction to access acquired advanced planning skills, along with an increase in writing instructional time to enhance their genre and topical background knowledge. In a similar vein, Ritchey et al. (2015) explored the relationship between teachers' orientations and writing instructional practices, which varied by grade level, with older students producing superior texts and their teachers adjusting their instructional foci according to their students' developing competencies. Additionally, Coker et al. (2018) examined the connections between generative writing instruction and student achievement, which were found to vary based on two student factors (i.e., ethnicity and gender). Specifically, male and minority group students displayed higher writing quality than their counterparts when exposed to increasingly generative writing practices. Taken together, these studies provide critical implications for educators and researchers, emphasizing the need to address the ways in which student variables interact with influential teacher variables to facilitate student learning and construct classroom contexts.

## 5. Research objectives for this study

**Figure 1:**
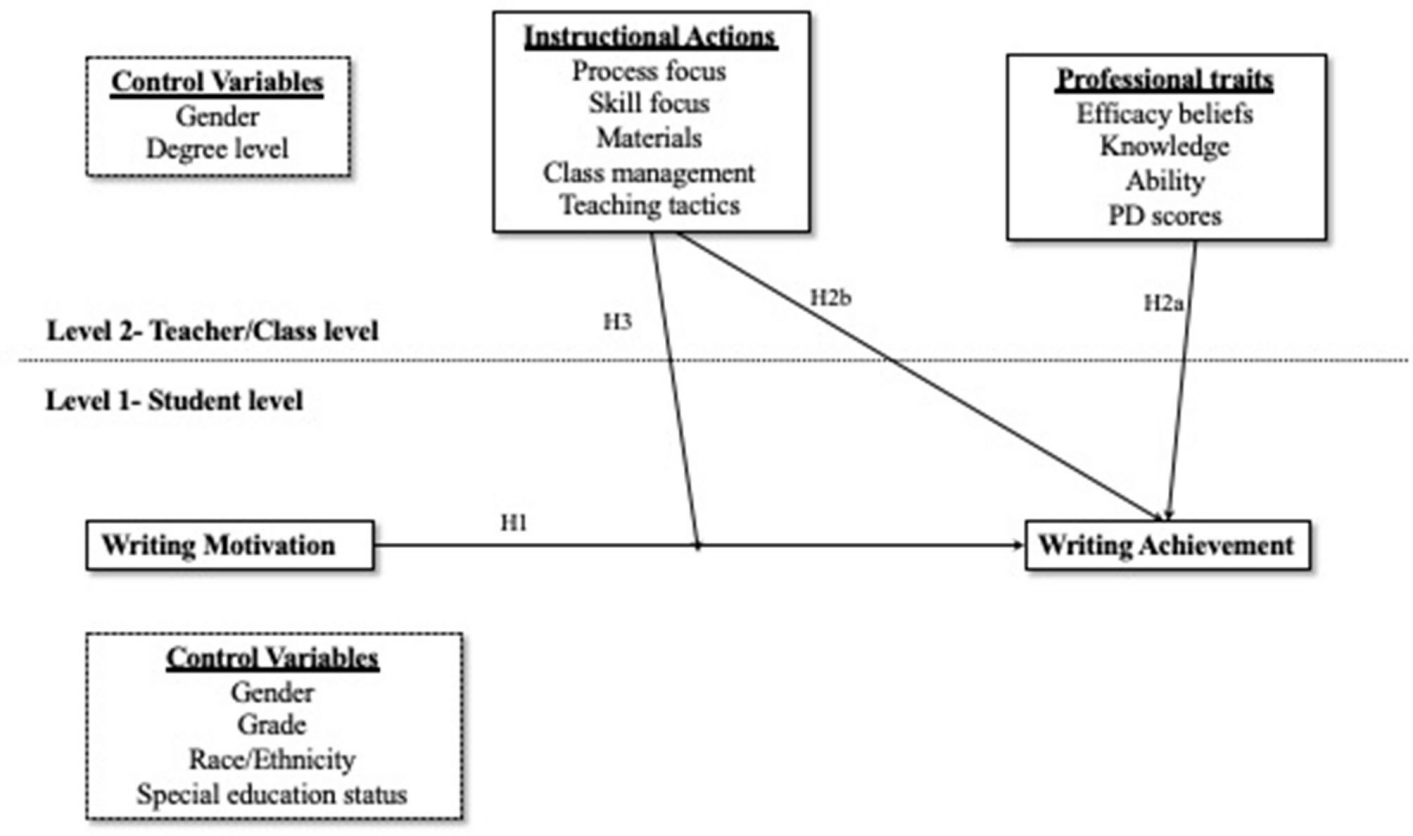
The proposed conceptual model among variables for the multilevel analysis.

Although prior research has shed light on the factors that influence student writing achievement, there remain gaps in our understanding of how these factors interact and influence student writing outcomes. Specifically, exploring the interplay between student motivational beliefs, teacher professional traits, teacher instructional practices, and student writing achievement holds promise to inform the development of effective interventions that promote and sustain writing development. This study aims to expand on previous research by examining the relationships among these variables in upper elementary students using hierarchical linear modeling. The proposed conceptual model is presented in Figure 1. The study addresses the following research questions and corresponding hypotheses as follows:

*Research Question 1:* Does students' writing motivation predict their writing quality?
*Hypothesis 1:* Students' writing motivation relates to their writing quality. More specifically, we posited that the composite motivational scores of students, encompassing self-efficacy beliefs, task interest and value, and outcome and efficacy expectations, would exert a positive influence on their writing performance.
*Research Question 2:* Do teachers' professional traits and teaching effectiveness predict students' writing quality?
*Hypothesis 2a:* Teachers' self-efficacy beliefs, writing knowledge, writing ability, and professional development efforts relate to students' writing quality.
*Hypothesis 2b:* Teachers' instructional practices related to process, skills, materials, teaching tactics, and classroom management relate to students' writing quality.
*Research Question 3:* Does the relationship between students' writing motivation and their writing quality depend on teachers' instructional practices?
*Hypothesis 3:* Teachers' instructional practices related to process, skills, materials, teaching tactics, and classroom management moderate the relation between students' writing motivation and writing quality.

## 6. Method

### 6.1. Participants and setting

The present study is a subset of a larger research project that aimed to evaluate changes in students' writing motivation, knowledge, and performance over a school year, disaggregated by genres at multiple levels of analysis, including district, classroom, teacher, and student levels (see Troia et al., 2020). The sample data analyzed in this study were obtained from 41 English language arts teachers from 18 suburban districts in the Midwestern United States. A total of 346 students were selected based on their writing ability levels, as determined by either their district writing assessment scores or their teacher's ratings of the quality of their beginning-of-year writing samples evaluated using a common rubric in the district. Students varied between low, average, and high writing performance based on this information. The dataset was organized using a two-level stratified cluster sampling design, with students as the first level and teacher/classroom as the second level. As such, the findings from this study can be generalized to similar populations, as the sample included a diverse range of writing ability levels.

### 6.2. Student instruments

#### 6.2.1. Demographics

At the beginning of each school year, the participating teachers provided students' sociodemographic information, including their grade level, gender, race/ethnicity, and disability status through a survey. When data collection began, the students self-identified their gender and race/ethnicity on a participant information form. Of the 346 students, 46.5% (*n* = 161) were fourth graders, 55.5% (*n* = 192) were female, 72.0% (*n* = 249) were White, and 7.8% (*n* = 27) were students who received special education services.

#### 6.2.2. Writing motivational scale

The Situated Writing Activity and Motivation Scale (SWAMS) is a self-report tool used to measure students' motivation levels across three writing genres: narrative, informative, and persuasive. Based on an earlier version developed by Troia et al. (2013), the SWAMS consists of 15 Likert-scale items rated on a 7-point scale (ranging from 0 representing *strongly disagree* to 6 representing *strongly agree*) for each genre that measure three common motivational constructs of writing: self-efficacy beliefs, task interest and value, and outcome and efficacy expectations. Confirmatory factor analyses were performed to determine the factorial structure of the motivational instrument. The results revealed that a single motivation factor using all 15 items was sufficient to represent writing motivation in each genre, with good internal consistency reliabilities (Cronbach's α ranged from 0.85 to 0.87). The model also exhibited good fitness, as evidenced by CFI = 0.97 and RMSEA = 0.073. Furthermore, significant correlations have been observed between the motivation for narrative, informative, and persuasive writing (see Troia et al., 2022; **Table 2**), indicating strong associations ranging from 0.89 to 0.90. Therefore, to represent students' overall level of writing motivational beliefs across three genres, a composite score was computed in this study by averaging the three genre-specific writing motivation scores.

#### 6.2.3. Writing quality

Over the course of the academic year, students were required to complete four writing tasks for each of the three genres: narrative, opinion, and informative. These tasks were administered through an online writing assessment tool (see Truckenmiller et al., 2020), with each genre containing four distinct prompts that were presented in a counterbalanced order. To assess the quality of the students' typewritten responses, two trained research assistants utilized an analytic trait scoring rubric based on the Smarter Balanced Assessment Consortium writing rubrics (Troia et al., 2020). The raters evaluated the quality based on seven dimensions, including orienting the reader to the purpose of the text, grouping ideas to enhance text coherence, providing a concluding sentence or section, linking ideas using transition words and phrases, developing ideas with facts, examples, experiences, and descriptive details, using varied and appropriate language and vocabulary, and using correct grammar, usage, and mechanics. Each dimension was double scored on a scale of 0 to 5, resulting in a total score ranging between 0 and 35. To ensure interrater reliability, a two-way random effects intraclass correlation with absolute agreement was calculated, yielding coefficients of 0.80, 0.81, and 0.84 (Troia et al., 2022). Similar to the findings regarding writing motivation, our study revealed statistically significant correlations among the writing quality of three distinct genres (see Troia et al., 2022; **Table 2**), demonstrating correlation coefficients ranging from 0.81 to 0.85. These results indicate moderate to strong associations between three assessed writing qualities. In order to determine overall writing quality, a composite score was calculated by taking the average score of all essays completed by each student.

### 6.3. Teacher instruments

#### 6.3.1. Demographics

The study involved 41 teachers who taught fourth and fifth grade English language arts classes. The majority of the participating teachers were female (95.1%) and White (92.7%), but the sample also comprised two African American teachers and one Asian American teacher. Of the 41 participating teachers, 10 (24.6%) held only a bachelor's degree, and 20 (48.8%) taught fourth grade classes. On average, the teachers were 41.59 years old (SD = 1.45, range = 26–61). They had an average of 15.01 years of teaching experience and reported an average of 6.64 years of teaching fourth or fifth grade writing classes, depending on the grade level they were currently teaching when data collection was conducted.

#### 6.3.2. Self-efficacy beliefs

The Teacher Efficacy for Writing Scale (TEWS) is a self-report instrument originally developed by Graham et al. (2001). In the present study, the scale was modified by excluding eight items related to assessing teachers' general teaching efficacy factor, as these items exhibited low internal consistency reliability. The TEWS utilized in this study is composed of eight questionnaire items that assess teachers' perceived competence in teaching writing, using a six-point scale that ranges from strongly disagree to strongly agree (total scores ranging from 8 to 48; Cronbach's α = 0.84, CFI = 0.92). A higher mean score across items indicates greater teacher efficacy. The TEWS questionnaire items pertain to asking teachers' abilities to implement effective strategies for teaching writing, enhance student retention of introduced concepts, teach writing concepts and skills for rapid mastery, assist students with their most challenging writing problems, adjust the difficulty of writing assignments for struggling students, accurately assess the reasons for a student's writing difficulties, provide appropriate accommodations, and manage disruptive behaviors during writing time. Overall, the TEWS serves as a valuable means of gauging teachers' perceptions of their writing instruction efficacy. The average score of the eight items was used in this study to represent teachers' self-efficacy beliefs.

#### 6.3.3. Writing knowledge

The Teaching Writing Knowledge Test (TWKT) is an assessment tool aimed to measure teachers' writing content knowledge and pedagogical content knowledge. TWKT encompasses a total of 32 questionnaire items with 116 unique multiple-choice or fill-in responses scored as correct or incorrect (total score ranging from 0 to 116). The test includes items from research-based spelling and grammar knowledge tests for teachers (e.g., Cajkler and Hislam, 2002; Myhill et al., 2013), as well as items from other available tests used to evaluate pedagogical content knowledge of teachers (e.g., Cambridge English Teaching Knowledge Test). The TWKT also incorporated original items developed by the researchers. The test evaluates teachers' knowledge of key writing concepts such as morphemes, phonemes, syllables, consonant and vowel digraphs, consonant blends, root words, derivational and inflectional suffixes, regular and irregular spelling patterns, parts of speech, sentence structure, writing mechanics (capitalization, punctuation, and spelling), genre traits, evidence-based writing instruction practices, and targeted instructional activities to address various aspects of writing. The instrument has an internal consistency reliability of 0.72.

#### 6.3.4. Writing ability

The participating teachers were asked to undertake two subtests of the Wechsler Individual Achievement Test-Second edition (WIAT-II; Wechsler, 2005) at the beginning of the school year. These subtests, which evaluated the teachers' spelling and written expression skills, yielded standardized age-based scores as measures of writing proficiency. As one of our primary outcome variables at the teacher-level, a composite score was derived by tallying the standardized scores of the two subtests to represent teachers' overall proficiency in writing.

#### 6.3.5. Professional development

A researcher-designed questionnaire of three items is used to assess the nature of teachers' pre-service and in-service professional development (PD) opportunities related to teaching writing. The first item asks about the number of pre-service courses that included information on writing instruction, with response options ranging from 0 (none) to 2 (two or more) or those that were fully devoted to writing instruction, with response options ranging from 3 (one) to 4 (two or more). The second item focuses on the number of in-service activities related to writing instruction that teachers had participated in over the prior 5 years, which included live or online workshops, as well as formal or informal coaching/mentoring activities, with options ranging from 0 (none) to 4 (more than 6). The third item aims to capture the extent of teachers' unique independent learning activities to improve their writing instruction skills, such as engaging in more writing, reading about effective writing instruction, observing other teachers' writing instruction, seeking feedback on their writing instruction, and participating in additional courses or workshops not part of preservice or in-service training. The response options for this item ranged from 0 to 5. The total score for the questionnaire ranged from 0 to 13.

#### 6.3.6. Instructional practices observation

Over the course of the academic year, beginning in October/November and ending in April/May, the writing classes of the participating teachers were observed typically four times. It is worth noting that unforeseen disruptions, particularly during the COVID-19 pandemic, occasionally impeded the researchers' attempts to maintain a consistent interval between observation points. The observers received extensive training in project meetings before conducting the observations and employed a time-sampling procedure to document the occurrence of assigned instructional practices within each 10-min interval. To record the instructional practices, the two observers used iSeeNCode (Hofstetter, 2016), an iPad application with 131 binary codes (0 = absent, 1 = present) derived from the Observation Protocol for Writing Assessment and Instruction (OPWAI). The OPWAI was subdivided into eight major observation dimensions: (1) grouping, (2) process feature focus, (3) genre focus, (4) product feature focus, (5) materials, (6) instructional tactics, (7) management tactics, and (8) assessment. The present study places a particular emphasis on five dimensions of writing instruction, including process feature focus, product feature focus, materials, instructional tactics, and management tactics. To represent each dimensional code, the average proportion of relevant codes to the total number of observation codes (131) per observation segment was calculated across all observations. A higher value for each dimensional code indicates that teachers exhibited a greater frequency of taking actions related to that particular dimension during their observed classes. This approach allows for a quantitative assessment of the extent to which teachers implemented instructional practices related to the five dimensions of writing instruction examined. The components and subcomponents that were encompassed within the five dimensions, along with the interobserver agreement reliability statistics for each dimension, are displayed in the Supplemental material.

### 6.4. Data analysis strategy

Our study utilized a multilevel structure dataset comprised of 346 students nested within 41 classrooms. This hierarchical structure implies that a student's learning outcome is influenced by both their individual characteristics and the broader class environment. Since simple regression is not suited for analyzing nested data due to the assumption of independence among observations, we employed hierarchical linear modeling (HLM) as our major statistical approach. HLM allows for the accommodation of the nested structure and parameter estimation of the effects of predictors at different levels simultaneously. Given the large sample size within clusters in our case, we also employed the maximum likelihood estimation (MLE) method for accurate parameter estimation on fixed and random effects (Maas and Hox, 2005). Due to the sensitivity of HLM to missing data at level 2 or above, we removed 2 instances of missing data at the teacher level, resulting in a final sample of 41 eligible teachers. All HLM analyses were conducted using lmer package in R Studio 2023.03.0.

During the exploratory phase of our study, we used descriptive and correlational analyses to determine potential covariates and gain preliminary insights into our data characteristics prior to model estimation. However, we acknowledge the limitations of solely relying on correlational analyses as they were indicative of interdependence rather than causality and did not account for correlations across multiple levels. Thus, in the subsequent stages of our study, we adopted HLM analyses to uncover the distinct main and interaction effects of the study measures across different levels.

The present study employed a linear two-level HLM model with MLE method to explore the complexity of the outcome variable of student writing quality. The analytical procedure involved four major steps. Firstly, a null model with no independent variables at both student and teacher levels was executed to assess the proportion of variance in student writing quality that can be attributed to differences between classrooms in addition to the magnitude of variance within classrooms. The intraclass correlation coefficient (ICC) was computed to summarize the proportion of total variance in student writing achievement that is attributable to differences between classrooms. If the ICC value is >0.058, the differences across groups cannot be neglected and should be explained using more complex models (Cohen, 1988).

Secondly, a level 1 model was employed by incorporating student-level variables, including writing motivation as the principal student-level predictor, along with four relevant covariates (i.e., gender, grade, race/ethnicity, special education status). The level 1 model was designed to examine the effects of student-level predictors on student writing quality.

Thirdly, a level 2 model incorporating teacher-level variables was fitted to test the effects of these variables on student writing quality while accounting for the effects of teacher covariates. The teacher-level variables were categorized into two dimensions. The first dimension consisted of personal and professional attributes of a given teacher, including self-efficacy, writing knowledge, writing ability, and professional development score. The second dimension pertained to teacher instructional effectiveness, focusing on process features, product features, materials, teaching tactics, and class management. By controlling for two demographic covariates, namely gender and educational attainment (i.e., degree), the level 2 model analysis aimed to unpack the unique contribution of teacher-level factors to student writing quality.

Finally, a full model was conducted to examine the cross-level moderator effect. Specifically, the interaction terms between instructional actions at the teacher level and writing motivation at the student level were of primary interest in this study, while six covariates at both student and teacher levels were also included in the full model to control for their potential influence. To ensure accurate and unbiased estimates of the relationship between variables in our HLM, we utilized a strategy of centering variables. Specifically, all student-level variables were centered on the grand mean, while all teacher-level variables were centered on the group mean. This adjustment allowed for easier interpretation of the fixed effect of the level 1 predictor, improved the convergence of the model, and helped to avoid issues of multicollinearity in models with interaction terms. This approach is supported by prior research (Raudenbush and Bryk, 2002; Hayes, 2006) and is a common practice in hierarchical linear modeling.

The full model can be mathematically presented as follows:


$$\begin{array}{l} Writing\;achievement\;\left(of\;{individual}_{i}\in {class}_{j}\right)=\gamma_{00}+\gamma_{10}\left(gender\right)\\ +\gamma_{20}\left(race\;or\;ethnicity\right)+\gamma_{30}\left(grade\right)+\gamma_{40}\left(special\;education\;status\right)\\ +\gamma_{50}\left(motivation\right)+\gamma_{01}\left(gender\right)+\gamma_{02}\left(degree\right)+\gamma_{03}\left(Efficacy\right)\\ +\gamma_{04}\left(knowledge\right)+\gamma_{05}\left(ability\right)+\gamma_{06}\left(PD\right)+\gamma_{07}\left(process\;focus\right)\;\\ +\gamma_{08}\left(material\right)+\;\gamma_{09}\left(teaching\;tactics\right)+\gamma_{010}\left(class\;management\right)\\ +\gamma_{011}\left(skill\;focus\right)+\gamma_{11}\left(motivation\times process\;focus\right)\\ +\gamma_{12}\left(motivation\times material\right)+\gamma_{13}\left(motivation\times teaching\;tactics\right)\\ +\gamma_{14}\left(motivation\times class\;management\right)+\gamma_{15}\left(motivation\times skill\;focus\right)\\ +r_{ij}+\;u_{oj} \end{array}$$

where:

*r*_*ij*_= random effect for student *i* in classroom *j*;

*u*_*oj*_= random effect for classroom *j*.

HLM models can be evaluated using multiple criteria. The model fit comparison analyses were conducted using one-way ANOVA. The Akaike information criterion (AIC) and Bayesian information criterion (BIC) are commonly used fit indices, where lower values indicate superior model fit. The deviance statistic is another measure of fit for the covariance components of a model, which is calculated as −2 times the log likelihood function. Lower deviance values indicate a better model fit. Additionally, the difference in deviance statistics between two nested models can be used to test the hypothesis of whether additional predictors can improve model fit (Jayetileke, 2021). The difference in statistics follows a chi-square distribution, with degrees of freedom equaling the difference in the number of estimated parameters in the covariance component of the two models (Davison et al., 2002). These criteria are essential in evaluating the adequacy of HLM models and ensuring that the model accurately captures the relationship between variables.

## 7. Results

### 7.1. Exploratory data analysis

Table 1 presents the descriptive statistics of the student and teacher measures. Tables 2, 3 present unadjusted bivariate correlations for within-group (student) and for between-group (teacher) measures, respectively. At the student level, student demographic variables, including gender, grade, race/ethnicity, and disability status, were significantly correlated with their writing motivation and quality. Specifically, the positive and moderately strong correlation (*r* = 0.362, *p* < 0.01) between grade and paper quality suggested that moving from the grade 4 category to grade 5 category was moderately associated with an increase in quality, Gender was also found to have a positive but relatively low magnitude association (*r* = 0.117, *p* < 0.05) with paper quality, suggesting that moving from the male category to female category was associated with an increase in paper quality. Race/ethnicity showed a negative correlation (*r* = −0.117, *p* < 0.05) with writing motivation, indicating that moving from White category to non-White category was associated with a decrease in writing motivation. Disability status was found to be significantly associated with both motivation (*r* = −0.177, *p* < 0.01) and quality (*r* = −0.291, *p* < 0.05), suggesting that students with disabilities tended to demonstrate lower writing motivation and paper quality than typically achieving students. Therefore, these demographic variables were incorporated as covariates in subsequent HLM analyses.

**Table 1:** Descriptive statistics and coding for the student and teacher measures included in the model.

**Variable**	***n* (%)**	***M* (SD)**	**Range**
**Student level**			
**Gender**			
Female (coded as 0)	192 (55.5%)		
Male (coded as 1)	154 (44.5%)		
**Grade**			
Grade 4 (coded as 0)	161 (46.5%)		
Grade 5 (coded as 1)	185 (53.5%)		
**Race/Ethnicity**			
White (coded as 0)	249 (72.0%)		
Non-White (coded as 1)	97 (28.0%)		
**Disability status**			
Typically developing students (TD; coded as 0)	319 (92.2%)		
Students with disability (SWD; coded as 1)	27 (7.8%)		
Motivation		4.465 (0.821)	1.923–5.887
Writing quality score		13.659 (4.115)	3.833–23.833
**Teacher level**			
**Gender**			
Female (coded as 0)	39 (95.1%)		
Male (coded as 1)	2 (4.9%)		
**Degree**			
Bachelor (coded as 0)	10 (24.4%)		
Master (coded as 1)	31 (75.6%)		
Efficacy		4.182 (0.561)	2.75–5
Knowledge		89.561 (10.288)	62–107
Ability		230.926 (14.771)	194–256
Professional development (PD)		5.634 (2.904)	1–13
**Instructional practices**			
Process focus		1.211 (0.368)	0.44–3
Material		3.381 (0.783)	1–4.86
Teaching tactics		6.320 (0.832)	4.83–8.61
Class management (CM)		1.394 (0.857)	0–4.75
Skill focus		1.308 (0.276)	1–1.89

**Table 2:** Bivariate correlations for within-group (student) measures.

**Variable**	**1**	**2**	**3**	**4**	**5**	**6**
1. Gender	1					
2. Grade	0.225^**^	1				
3. Race/Ethnicity	−0.094	−0.024	1			
4. Disability	0.024	0.012	0.010	1		
5. Motivation	−0.107	−0.014	−0.117^*^	−0.177^**^	1	
6. Quality	0.117^*^	0.362^**^	−0.100	−0.291^**^	0.421^**^	1

Spearman/point-biserial correlation coefficient for categorical variables 1–4 and Pearson correlation coefficient for continuous variables 5–6.

^**^p < 0.01.

^*^p < 0.05.

**Table 3:** Bivariate correlations for between-group (teacher) measures.

**Variable**	**1**	**2**	**3**	**4**	**5**	**6**	**7**	**8**	**9**
1. Gender	1								
2. Degree	−0.128^*^	1							
3. Efficacy	−0.050	0.109^*^	1						
4. Knowledge	0.121^*^	0.080	0.128^*^	1					
5. Process	−0.130^*^	−0.160^**^	−0.114^*^	−0.338^**^	1				
6. Material	−0.081	0.188^**^	0.003	0.113^*^	−0.367^**^	1			
7. Teaching	0.022	−0.111^*^	−0.010	0.021	0.380^**^	−0.222^**^	1		
8. CM	−0.188^**^	−0.023	−0.224^**^	−0.217^**^	−0.242^**^	−0.026	−0.102	1	
9. Skill	−0.137^*^	0.137^*^	0.260^**^	−0.240^**^	−0.051	0.123^*^	−0.052	0.254^**^	1

Spearman/point-biserial correlation coefficient for categorical variables 1–2 and Pearson correlation coefficient for continuous variables 3–9.

^**^p < 0.01.

^*^p < 0.05.

At the teacher level, teachers' gender and degree information displayed significant associations with other teacher variables. For instance, the weakly positive correlation (*r* = 0.121, *p* < 0.05) between gender and teacher writing knowledge indicates that moving from the female category to male category weakly corresponds with an increase in their writing knowledge, or higher writing knowledge tends to co-occur with the male category. In addition, the weakly positive correlation (*r* = 0.109, *p* < 0.05) between degree and teacher efficacy implies that moving from teachers with bachelor's degrees to teachers with master's degrees was weakly associated with an increase in their teaching efficacy beliefs, or higher efficacy beliefs tend to co-occur with teachers with a master's degree. This finding is consistent with other studies (e.g., Yilmaz and Çokluk-Bökeoglu, 2008; Orakci and Karagöz, 2023) suggesting that as teachers' level of education progresses, they are likely to develop a more profound comprehension of writing, which may enhance their efficacy beliefs regarding their own writing skills and their effectiveness in teaching writing. Hence, the effects of teacher's gender and degree variables were controlled in the subsequent analyses.

### 7.2. Unconditional model

**Figure 2:**
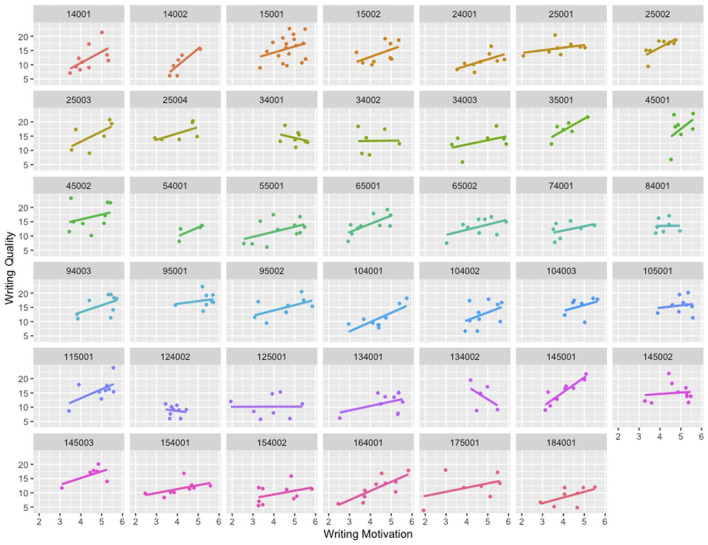
Student writing motivation, quality scores, and their relational slopes disaggregated by teacher/classroom. Each box in the figure represents a unique teacher ID (*n* = 41).

To estimate the extent to which writing achievement varied at the student and teacher levels, we initiated our HLM analysis by conducting a one-way random-effects ANOVA model, also referred to as an unconditional model, and included the dependent variable of writing quality as the sole factor. The intercept was found to be significant at 13.66, *t*_(38)_ = 36.59, *p* < 0.001, representing the overall average score of writing quality without any predictors in the model. The intraclass correlation coefficient (ICC) was 0.24, indicating that a considerable proportion (i.e., 24%) of the variance in student writing quality could be attributed to differences between classrooms, whereas most of the variance was due to differences between students. As our ICC was above the conventional threshold (i.e., 0.058; Cohen, 1988), further analyses were required to explain the variance related to differences between teachers and students. The ICC result also revealed the nested data structure of this study, making HLM an appropriate approach for examining our data. Furthermore, Figure 2 displays students' writing motivation and quality scores within each class, reinforcing the nested nature of the data and the necessity for multilevel modeling analysis. The varying slopes depicted in Figure 2 indicate that the factors contributing to the variability between classrooms needs to be explained in the subsequent models. The HLM results are given in Table 4.

**Table 4:** Results from HLM predicting student writing quality scores.

**Parameter**	**Unconditional**		**Student-level**		**Teacher-level**		**Full**	
	**Coeff**.	**SE**	**Coeff**.	**SE**	**Coeff**.	**SE**	**Coeff**.	**SE**
**Fixed effects**								
Intercept γ_00_	13.66^***^	0.37	12.94^***^	1.14	11.77	0.89	8.99^*^	3.82
**Level 1: student**								
Gender γ_10_			−1.02^**^	0.35	−1.02^**^	0.35	−1.04^**^	0.36
Race/Ethnicity γ_20_			−0.25	0.42	−0.31	0.42	−0.22	0.44
Grade γ_30_			2.98^***^	0.60	2.46^***^	0.63	2.46^***^	0.67
SPED γ_40_			−3.81^***^	0.65	−3.91^***^	0.65	−4.03^***^	0.66
Writing motivation γ_50_			1.54^***^	0.23	1.53^***^	0.23	0.82	2.59
**Level 2: teacher**								
Gender γ_01_					0.77	1.17	0.76	1.44
Degree γ_02_					0.59	0.63	0.59	0.77
Efficacy γ_03_					−0.26	0.57	−0.27	0.69
Knowledge γ_04_					−0.04	0.03	−0.04	0.04
Ability γ_05_					−0.04	0.02	−0.04	0.03
PD γ_06_					0.10	0.09	0.10	0.11
Process focus γ_07_					−0.57	0.93	−0.59	1.15
Material γ_08_					0.06	0.35	0.05	0.43
Teaching tactics γ_09_					0.66^*^	0.33	0.67	0.40
Class management (CM) γ_010_					−0.74^*^	0.35	−0.76^*^	0.43
Skill focus γ_011_					0.40	1.03	0.43	1.25
**Cross-level interactions**								
Motivation × process γ_11_							−1.27^*^	0.69
Motivation × material γ_12_							−0.60^*^	0.33
Motivation × teaching γ_13_							0.59^*^	0.30
Motivation × CM γ_14_							−0.26	0.27
Motivation × skill γ_15_							0.74	0.91
**Random effects**								
Level-1 effect *r*_*ij*_	12.75		9.09		9.09		9.10	
Classroom mean *u*_**o***j*_	4.13		2.50		1.17		2.30	
ICC	0.24		0.21		0.11		0.20	
Between-classroom variance explained (%)	NA		32		40		38	
Within-classroom variance explained (%)	NA		14		6		12	
**Goodness-of-fit**								
AIC	1,922.1		1,810.2		1,813.4		1,815.3	
BIC	1,933.7		1,840.9		1,886.5		1,907.7	
Log Likelihood	−958.1		−897.08		−887.7		−883.7	
Deviance	1,916.1		1,794.2		1,775.4		1,767.3	
Chi-square (df)			121.9 (5)^***^		18.7 (11)^*^		8.1 (5)	

^*^p < 0.10.

^**^p < 0.05.

^***^p < 0.01.

### 7.3. Level 1 model: student-level

The level 1 model was employed to investigate the associations between students' writing motivational beliefs and writing quality while holding the four covariates constant. Results from the level 1 model supported our proposed Hypothesis 1 that student writing motivation had a positive effect on their writing quality, with a one-scale point increase in writing motivation resulting in a 1.61-point increase in writing quality. Moreover, the results revealed that students who were in fifth grade, female, and typically achieving had significantly better writing performance than their counterparts.

Incorporating student-level predictors into the model accounted for ~32% of the between-class variance in writing quality, while the estimated within-class variance decreased from 0.24 in the unconditional model to 0.14 in the student-level model. The reduction in within-class variance suggested that the addition of student-level predictors was not able to account for a significant portion of the within-group variability in writing achievement, and/or there may be other unmeasured factors that were influencing writing achievement at the student level. Furthermore, based on the model fit comparison (see Table 4), the resulting level 1 model demonstrated a significantly better goodness of fit [χ^2^(5) = 121.96, *p* < 0.001] than the unconditional model, indicating that the integration of student-level predictors significantly improved the model's ability to explain the variance in writing quality.

### 7.4. Level 2 model: teacher-level

The level 2 model was utilized to further explore the factors that influence student writing achievement by adding teacher-level predictors based on personal and professional attributes, as well as instructional actions. After controlling for two demographic covariates (namely gender and degree), our analysis revealed that, while teacher personal and professional characteristics did not significantly affect student writing quality, there were some notable effects observed between teacher instruction and student writing performance. Specifically, the use of effective teaching tactics, such as modeling, explanation, summarizing, and questioning, had a positive impact (γ = 0.66, *p* < 0.10) on student writing quality, while the frequent use of class management strategies had a negative effect (γ = −0.74, *p* < 0.10). Our findings highlighted the importance of effective teaching practices in shaping student writing quality. Effective teaching strategies, such as giving clear writing directions, facilitating discussions about writing-related issues, and using questioning techniques to gauge understanding, can enhance student writing performance. Conversely, instructional strategies that aim to monitor, support, alter, or control student writing behaviors may impede student writing achievement to some extent.

It is important to acknowledge that we applied a less stringent criterion for significance testing (i.e., *p* < 0.10) to interpret the results. The decision was made with the aim of increasing the likelihood of detecting interaction effects that hold theoretical importance while mitigating the risk of overfitting, which can arise when including too many variables in a model with a limited sample size (Scherbaum and Ferreter, 2009). Moreover, a significance level of 0.10 also was utilized to interpret the interaction results in the subsequent analyses. It is crucial to recognize that this approach introduces a limitation to the study.

By incorporating the main effects of teacher-level predictors, our level 2 model demonstrated an improved capability to account for 40% of the between-class variance in student writing achievement, resulting in a decrease in the estimated within-class variance by 0.08. Comparing the level 2 model to the level 1 model, level 2 model exhibited a better goodness of fit, as evidenced by its decreased deviation value of 1775.4 and a higher fit statistic [χ^2^(11) = 18.754, *p* = 0.06]. These findings suggest that the level 2 model is more effective in predicting data and provides a more accurate representation of the factors that impact student writing achievement.

### 7.5. Full model: moderating effect of teacher's instructional practices

Finally, a full model with multiple cross-level interaction terms was used to examine the joint effects of students' motivational beliefs and teachers' writing instructional practices on writing achievement. The findings showed that, at the student-level, gender, grade, and disability status remained significant predictors of writing quality, whereas the main effect of student motivational beliefs was no longer significant. However, we indeed found that student motivational beliefs had weak but significant interaction effects when combined with other writing instructional practices variables. This suggested that the effect of student writing motivation may be modified by other variables with which it interacted in a more complex model, such as teachers' implementation of certain writing instructional practices.

The findings indicated that the interaction term between student motivation and teacher instruction on process features was marginally significant and negative (γ = −1.27, *p* < 0.1), indicating that the relationship between student motivational beliefs and their writing achievement was moderated by the frequency of teacher instruction on process features. Specifically, the negative effect of student motivation on their writing achievement was found to be marginally significantly stronger when teacher instruction on process features was more frequent, compared to when it was less frequent. The observed decrease in the scale of the effect was weakened by a value of 1.27 units.

The interaction term of motivation × materials was also marginally significant and negative (γ = −0.60, *p* < 0.1), suggesting that the relationship between student motivational beliefs and their writing achievement was moderated by the more frequent use of materials in writing class. Specifically, the negative impact of student motivation on their writing achievement was found to be marginally significantly stronger when the frequency of utilizing materials in the writing class was higher compared to when it was lower. The observed decrease in the scale of the effect was weakened by a value of 0.60 units.

Conversely, the interaction term between student motivation and the frequency of utilizing teaching tactics in the writing class was marginally significant and positive (γ = 0.59, *p* < 0.1), indicating that the relationship between student motivational beliefs on writing and their writing achievement was moderated by the frequency of employing teaching tactics in the writing class. Specifically, the positive effect of student motivation on their writing achievement was found to be more evident when there was increased frequency of utilizing teaching tactics in the writing class compared to when it was lower. The observed increment in the scale of the effect was increased by a value of 0.59 units.

The full model, which included five pairs of interaction terms, did not significantly improve the fit of the model compared to the level 2 model, as indicated by the ANOVA chi-square test χ^2^(5) = 8.066, *p* = 0.15. In other words, the difference in fit between the level 2 model and full model is not statistically significant. While this non-significant result may suggest issues with statistical power or small sample size, it is important to note that the additional predictors in the full model may still be important and meaningful in explaining the outcome variable. It is noteworthy that the full model showed a slightly lower capability in explaining between-class variance in student writing achievement compared to the level 2 model, with a decrease of 2%. However, the full model demonstrated an increase of 6% in its predictive ability for explaining variance in writing achievement within classrooms.

### 7.6. Summary of results

The results of bivariate correlational analyses and level 1 model, as presented in Tables 2–4, revealed that all student-level variables, except race/ethnicity, were significantly related to student writing achievement. However, only two teacher variables, namely teaching tactics and class management, exhibited significant effects on writing achievement but with different directional impacts, as demonstrated by the level 2 model results. The HLM analysis revealed that writing motivation had a positive predictive effect on writing achievement, as evident from significant results in both student- and teacher-level models.

Despite student motivation being non-significant in the final HLM analysis, our study identified significant interaction effects between motivational beliefs and instructional practices on writing achievement. Specifically, our findings suggested that students with high motivation were more likely to demonstrate better writing outcomes in a classroom setting where writing instruction emphasized fewer process features and materials but utilized more teaching tactics, compared to classrooms with the opposite characteristics. Table 5 provides a summary of the results our proposed hypotheses.

**Table 5:** Summary of hypotheses.

	**Hypotheses**	**Conclusion**
H1	Students' writing motivation relates to their writing quality	Supported
H2a	Teachers' self-efficacy beliefs, writing knowledge, writing ability, and professional development efforts on writing relate to students' writing quality	Not supported
H2b	Teachers' instructional practices related to of process focus, skills focus, materials, teaching tactics, and classroom management relate to students' writing quality	Partially supported
H3	Teachers' instructional practices related to process, skills, materials, teaching tactics, and classroom management moderate the relation between students' writing motivation and writing quality	Partially supported

## 8. Discussion and implications

Within the academic domain of writing, state content standards exert significant influence on guiding content and pedagogical approaches adopted by educators (Troia and Graham, 2016; Baez-Hernandez, 2019). Despite concerted efforts to incorporate a diverse array of writing task types and increase writing time across the curriculum, the impact of these standards on classroom instruction and subsequent writing outcomes may be curtailed due to the inadequate quantity and quality of writing practices provided throughout the United States (Graham et al., 2012). Additionally, the significant variability among teachers in terms of their experiences, values, beliefs, and attitudes toward writing proficiency and instruction poses a formidable challenge in implementing coordinated and effective writing instructional practices across diverse classrooms (Perry, 1998). This complexity necessitates a multifaceted approach when attempting to teach writing effectively and efficiently. Therefore, the aim of this study was to shed light on instructional practices and professional traits associated with writing that can promote students' motivation and performance. Our findings suggest an interrelated and integrated array of teachers' professional traits and instructional actions that can influence students' writing motivation and proficiency. Moreover, we observed that certain instructional practices targeting different aspects of developing students' writing performance can moderate the predictive power between students' writing motivation and their writing quality. Our findings not only validate students' writing strengths and weaknesses at the individual level, but also offer valuable insights for educators on implementing effective practices at the teacher level.

### 8.1. Student-level predictors of writing achievement

The outcomes of the student-level analysis indicated a significant association between students' motivational beliefs and their writing achievement, regardless of student demographics. Specifically, students who displayed a strong inclination toward writing, assigned value to producing multiple written products, and demonstrated confidence in their writing ability, tended to outperform in writing tasks compared to those who felt overwhelmed, frustrated, and lacked motivation toward writing. These findings were consistent with earlier research studies on writing motivation and achievement conducted by Pajares (2003), Graham et al. (2007), and Wilson and Trainin (2007), which also provided evidence of a significant positive correlation between writing motivation and achievement.

Furthermore, we explored the impact of students' sociodemographic characteristics on their writing achievement. Our analysis revealed that female students, fifth graders, and typically achieving students tended to produce higher quality writing than their male, fourth grade, and struggling counterparts. These findings aligned with prior research suggesting that gender (De Smedt et al., 2018), grade level (Shell et al., 1995), and learning ability (Troia and Graham, 2016) may have an impact on writing achievement, and should therefore be considered when designing writing instruction for elementary-aged children. Although the underlying reasons for these findings are not entirely evident, it is anticipated that girls, older students, and typically achieving students may have a more accurate understanding of their writing abilities, possess more advanced writing skills and strategies, set clearer goals for the writing process and product, and develop a theory of mind to understand their audience (Graham and Perin, 2007). Hence, students with these demographic characteristics are likely to develop into more advanced and sophisticated writers. Our results reinforce the notion that student motivational beliefs are malleable and can be influenced by various factors such as cultural background, personal interests, prior experience, and other individual traits (Pajares, 2003).

When examining the impact of student-level variables on writing achievement between classrooms, our study revealed that these factors accounted for a relatively lower proportion of the variance (i.e., 32%) compared to similar studies that employed multilevel analysis methods (e.g., Coker et al., 2018; Los and Schweinle, 2019) to explain writing outcomes. It is important to note that our study did not place primary emphasis on student-level factors, nor did we include other writing-related skills that have been found to significantly impact writing achievement, such as handwriting fluency, basic reading ability, and spelling, as was done in Coker et al.'s (2018) study. Future research could incorporate other student-related factors, such as writing knowledge and strategies, to capture a more comprehensive range of individual differences that may contribute to writing achievement.

The findings of our student-level analysis hold important implications for both preservice and in-service educators seeking to provide effective writing instruction for elementary-aged children. Firstly, along with considering the content and structure of the writing curriculum to benefit their students, it is also essential to consider individual student-level factors and tailor their instruction to meet the specific needs of each student to boost their motivation and writing achievement. To achieve this, educators should adopt a student-centered approach that acknowledges the social and cultural diversity of students' backgrounds and their unique motivational beliefs (see Land et al., 2012). Professional development opportunities should also be provided to educators to enhance their understanding of student motivation and effective writing instruction, particularly for students who are struggling or disengaged. By leveraging students' individual strengths and interests, educators can create a respectful, supportive, and engaging writing environment that fosters motivation and facilitates writing achievement for all students (Tucker, 2012).

Additionally, educators should consider providing targeted writing instruction and support for struggling students, including those who lack motivation or confidence in their writing ability, to help them overcome writing challenges and achieve writing success. This finding was also consistent with a prior study (Troia et al., 2022) that classified the same sample of students used here into five distinct written profiles, where motivation was identified as a critical writing-related measure that distinguished their profiles and further affected their writing quality in narrative, persuasive, and informative essays. To address the needs of unmotivated writers, instructional scaffolds with motivational elements, including self-regulatory activities to maintain motivation and individualize treatment (Zimmerman and Bandura, 1994) may be beneficial to keep students motivated and prevent them from falling behind.

### 8.2. Teacher-level predictors of writing achievement

While individual differences among students are undoubtedly significant contributors to the complexity of their writing achievement, it is essential not to overlook the impact of teacher/classroom-level factors in explaining the variance in writing achievement between classrooms. Our analysis revealed that teacher-level predictors significantly accounted for an additional 8% of the variance in explaining writing achievement beyond student-level factors.

In our study, we examined two dimensions of teacher-level factors. The first dimension of teacher-level factors was investigated, specifically the quantity of teaching practices across varied aspects of instruction. Our analyses revealed that teaching tactics were positively associated with student writing achievement, whereas class management was adversely related to student writing achievement. However, we did not observe any statistically significant impact on student writing achievement for other aspects of teacher actions. These findings suggest that the positive effect of teaching tactics on student writing achievement may be attributed to their ability to create a supportive and engaging learning environment through modeling, questioning, suggestions, feedback, and so forth, which can enhance student motivation and confidence in writing (Kapka and Oberman, 2001; Tienken and Achilles, 2003). On the other hand, excessive class management practices can disrupt student learning and negatively impact their motivation to write (Franklin and Harrington, 2019). Regarding the non-significant effects, it is possible that these effects were confounded by other factors. To explore this possibility further, we conducted a moderating analysis and found that some of the other teaching aspects had a significant impact on student writing achievement when motivation served as a moderator. The interacting relationships are discussed in a subsequent section.

Another domain involved investigating the impact of teachers' personal and professional traits on student writing achievement. However, we did not find any statistically significant effects of teacher degree, gender, efficacy beliefs, writing knowledge, writing ability, or professional development on student writing achievement. There are various reasons that could explain these findings. Firstly, our result was consistent with prior research that proved no significant relationship between teacher qualifications and student academic achievement (Huang and Moon, 2009; Kosgei et al., 2013). Secondly, the measures used to assess teacher-level factors in this study may not have been sensitive or specific enough to capture the nuances of these constructs. For example, self-efficacy beliefs are multifaceted and intricate constructions, and a narrow or insufficient measure may not be able to capture the full range of nuances in this construct. Similarly, for writing knowledge, we only analyzed teachers' writing ability in spelling and written expression using a standardized test (the WIAT-II), thereby neglecting the complex nature of this construct. Thirdly, it is also possible that teacher-level factors interact with other contextual factors; therefore, the effect of teacher-level factors may be masked or moderated by other factors. Hence, future study should investigate these contextual factors to obtain a more comprehensive understanding of the complex interplay between teacher-level factors and student writing achievement.

Our analysis of teacher-level factors has important implications. While we did not observe significant associations between teachers' personal and professional characteristics and student writing achievement, this does not necessarily imply that teachers should not strive to develop their own expertise and ability for teaching writing. Instead, we propose integrating these factors into a school district's accountability system can provide valuable empirical insights into the multifaceted process of teacher evaluation (see Kupermintz, 2003). Although it may be challenging to define the hallmarks of effective teachers, effective instructional practices can be identified and honed. When data on teacher effectiveness are coupled with professional development opportunities that concentrate on improving instructional characteristics and teaching behaviors, the ultimate result can be improved educational success for the majority of students (Stronge, 2006).

### 8.3. Moderating role of teachers' instructional effectiveness between student motivation and achievement

Our study has revealed three interaction effects at a significance level of 0.10. First, the interaction term of motivation × process was found to have negative impact on student writing achievement. This finding implies that, in classes where writing instruction on process features was infrequent, student motivation had a strong predictive effect on their writing achievement. It also can be interpreted that for students with lower writing motivation, providing writing instruction focused on process features was found to have a stronger predictive effect on their writing achievement; conversely, for students with higher writing motivation, such instruction may not provide as much benefit in facilitating their performance.^1^ This finding is in line with the notion that process-oriented instruction involves providing direct strategy instruction and scaffolded practice that integrates a set of theories, procedures, and activities into multiple writing processes such as planning, drafting, and revising. Previous research has suggested that such guided instruction can be effective in boosting writing performance and can be particularly beneficial for demotivated students (e.g., Collins, 1998; Lamb, 2017). Additionally, the literature also indicates that more experienced and mature writers typically use writing processes to compose essays, implying that motivated writers may have the capability to leverage their own self-regulation and may not derive as much benefit from guided instruction (e.g., Graham and Harris, 1996; Cleary and Zimmerman, 2004).

The second significant interaction effect we observed was between motivation and the utilization of writing materials during classes, which had a negative impact on student writing achievement. This suggests that in classes where writing materials such as graphic organizers, revision checklists, and word walls were infrequently utilized, student motivation had a strong predictive power on their writing achievement. In other words, for students with lower writing motivation, utilizing materials was found to have a stronger predictive effect on their writing achievement, whereas such teaching practices may not greatly profit students with higher writing motivation. This aligns with prior research that providing optimal learning materials can be engaging for unmotivated students and can provide additional support for their writing development with an effect size of 0.82 (see Graham and Perin, 2007).

Third, the interaction term of motivation × teaching tactics was found to positively influence student writing achievement. The result indicates that in writing classes where teaching tactics such as modeling, explanation, questioning, and conferencing/discussion were frequently employed, student motivation had a strong predictive effect on their writing achievement. Specifically, for students with high writing motivation, these instructional tactics were found to have a stronger predictive effect on their writing achievement, while for students with lower writing motivation, providing such instruction may not be as beneficial for facilitating their achievement. Effective and adequate teaching tactics can contribute to a positive learning environment and promote student engagement, ultimately leading to better academic performance. Our findings are consistent with previous research suggesting that teachers can establish positive relationships with the students and enhance their writing performance by providing varied instructional assistance, including modeling, demonstration, and discussion, as well as offering positive feedback and reinforcement for the use of writing strategies, and granting students more autonomy in selecting their writing topics (Troia et al., 2012; Bruning and Kauffman, 2016; Philippakos, 2020). Additionally, the finding that low-motivated students may not gain as much from teaching tactics is likely due to their lack of intrinsic drive and interest, which can hinder their ability to remain attentive and receptive during teacher-led instruction. To address this issue, educators may opt for incorporating instructional models such as the self-regulated strategy development approach, which involves teacher modeling followed by independent student practice and hands-on activities that have been demonstrated to promote creativity and boost student engagement (Harris et al., 2008). This approach provides opportunities for students to take an active role in their learning and apply concepts and strategies in meaningful and interactive ways, because relying solely on modeling and explaining strategies may prove inadequate for many students (Harris and Graham, 1999). It is also noted that the frequency of class management has a negative impact on student writing achievement in the full model, although this was not an interaction effect. Excessive classroom management activities may impede the time allocated for writing activities and disrupt teachers' planned instruction, leading to a shift in focus away from writing instruction (Marzano et al., 2003). In addition, the frequent use of punitive management strategies during class may decrease students' motivation to learn (Rahimi and Karkami, 2015). Previous research has shown that effective writing classes typically encounter disruptive behavior incidents approximately once every 2 h, while ineffective classes may experience such incidents as frequently as every 12 min (Stronge et al., 2007). It is crucial to maintain a balanced approach to class management that does not detract from writing instruction and avoids frequent disruptions that can interfere with students' learning.

## 9. Conclusions

Different from prior studies that relied on bivariate correlations or simple regression analyses to explore relationships, the present study addresses a major gap in the literature on cross-level effects by utilizing multilevel analysis within our nested dataset. We aimed to investigate how students with varying levels of motivation may benefit from specific teaching strategies to enhance their writing achievement. Future studies could expand upon our work by incorporating additional student-level predictors, which would allow for targeted instruction based on individual student characteristics. It is also important to acknowledge that our study primarily relied on quantitative observation data to examine the presence or absence of specific writing instructional actions employed by teachers in their writing class, rather than delving into the intricates of their implementation. For instance, we found a negative moderating effect of teaching materials on students' writing achievement. However, it is essential to recognize that simply providing students with tools or resources without adequate guidance may not positively moderate the relationship between motivation and performance. Conversely, if students are provided with the same tools along with the knowledge and skills to effectively utilize these materials, it might yield a positive moderating effect on the relationship between motivation and performance. Future research utilizing qualitative methods can offer a more nuanced exploration of the utilization of these instructional actions, allowing for a richer understanding of their effects on students' writing performance.

Although we did not find any significant effects of teacher personal or professional characteristics on student writing achievement, it is arbitrary to suggest that these factors are not important. In fact, teacher efficacy beliefs and writing expertise can enhance their effectiveness as both writers and educators, and may ultimately influence their instructional efficiency and promote a positive learning environment. Furthermore, our study emphasizes the importance of caution when implementing teaching tactics, given that students with varying levels of motivation may exhibit different levels of response to these instructional approaches. This finding has significant implications for educational practitioners, as it suggests the need for differentiated instruction that caters to the unique needs and characteristics of each student, to ensure that all students are engaged and motivated to learn.

## Data availability statement

The original contributions presented in the study are included in the article/Supplementary material, further inquiries can be directed to the corresponding author.

## Ethics statement

The studies involving human participants were reviewed and approved by Social Science Behavioral/Education Institutional Review Board, Michigan State University. Written informed consent to participate in this study was provided by the participants' legal guardian/next of kin.

## Author contributions

Conceptualization: HW and GT. Methodology, formal analysis, and writing—original draft preparation: HW. Writing—review and editing, project administration, and funding acquisition: GT. All authors have read and agreed to the submit the manuscript.

## Footnotes

^1^Interaction term indicates a bidirectional relationship. Therefore, we interpreted both directions of the interaction effects, whether motivation was the moderator or instructional practices were the moderator. The same approach for interpretation was taken for other interaction terms in this study. This allowed us to comprehensively explore the relationship between motivation, instructional practices, and writing outcomes.
