# Supplementary material for: How students' writing motivation, teachers' personal and professional attributes, and writing instruction impact student writing achievement: a two-level hierarchical linear modeling study
Source: Front Psychol. 2023 Jul 20;14:1213929. doi: 10.3389/fpsyg.2023.1213929 (PMC10398962; doi:10.3389/fpsyg.2023.1213929)
Supplement: Supplementary file 1 [file Table_1.docx]

Supplementary Material

How teachers’ personal and professional attributes and their writing instruction impact student writing achievement: A two-level hierarchical linear modeling study

Heqiao Wang*, Gary Troia

*** Correspondence:** Heqiao Wang: [wangheq2@msu.edu](mailto:wangheq2@msu.edu)

| **Dimension** | **Component** | **Subcomponent** | **Gwet AC** | |
| --- | --- | --- | --- | --- |
| **Process Feature Focus** | **Pre-writing** | **Topic/Genre selection**  **Mentor Text Examination**  **Gathering Information**  **Planning** | 0.80  0.90  0.86  0.76 | 0.67 |
|  | **Drafting** |  | 0.88 |  |
|  | **Revising** |  | 0.94 |  |
|  | **Editing** |  | 1 |  |
|  | **Publishing/Sharing** |  | 0.96 |  |
| **Skill Focus** | **Transcription** | **Spelling**  **Handwriting**  **Keyboarding** | 0.99  1  1 | 0.75 |
|  | **Conventions Usage** | **Capitalization**  **Punctuation** | 0.98  0.97 |  |
|  | **Grammar** | **Sentence Combining**  **Parts of Speech**  **Sentence Types** | 1  0.98  0.99 |  |
|  | **Vocabulary** | **Content**  **Traits**  **Style**  **Process**  **Genres** | 0.94  0.99  1  1  0.94 |  |
|  | **Text Structure** |  | 0.63 |  |
|  | **Paragraph Structure** |  | 0.79 |  |
|  | **Traits** |  | 0.76 |  |
|  | **Task/Audience/Purpose** |  | 0.65 |  |
|  | **Format Elements** |  | 0.74 |  |
|  | **Multimedia** |  | 1 |  |
| **Materials** | **Writing Notebook** |  | 0.63 | 0.85 |
|  | **Blackboard/Whiteboard/**  **Smartboard** |  | 0.79 |  |
|  | **Chart/Poster** |  | 0.76 |  |
|  | **Computer** | **Word Processing**  **Assistive Technology** | 0.84  1 |  |
|  | **Graphic Organizer** |  | 0.69 |  |
|  | **Revising/Edit Checklist** |  | 0.98 |  |
|  | **Word Wall/List** |  | 0.92 |  |
|  | **Loose Paper** |  | 0.57 |  |
|  | **Journal** |  | 1 |  |
|  | **Reference Resource** |  | 0.86 |  |
|  | **Rubric** |  | 1 |  |
|  | **Process Indicator** |  | 1 |  |
|  | **Source Text** | **Printed**  **Digital**  **Audiovisual** | 0.60  0.88  0.97 |  |
|  | **Revising/Edit Exercise** |  | 0.91 |  |
|  | **Evaluative Checklist** |  | 0.92 |  |
|  | **Workbook/Worksheet** |  | 0.78 |  |
|  | **Other** |  | 0.81 |  |
| **Teaching Tactics** | **Modeling** | **Using Classroom Literature**  **Using Generated Texts**  **Using Experiences**  **Using Materials**  **Using Think-Aloud**  **Using Other Instructional Tactics** | 0.76  0.61  0.95  0.85  0.96  0.99 | 0.84 |
|  | **Explanation** |  | 0.35 |  |
|  | **Conferencing/Discussion** | **Individual**  **Peer**  **Small Group**  **Whole Class** | 0.87  0.95  1  0.75 |  |
|  | **Questioning** |  | 0.87 |  |
|  | **Suggestions** |  | 0.57 |  |
|  | **Evaluative Statements/Feedback** | **Verbal Comments**  **Written Comments**  **Grade** | 0  0.96  1 |  |
|  | **Debriefing/Summarizing** |  | 0.96 |  |
|  | **Branching** |  | 0.55 |  |
|  | **Scribing** |  | 0.74 |  |
|  | **Collaborative Writing** |  | 0.88 |  |
|  | **Sustained Writing** | **Individual**  **Dyads**  **Small Group**  **Whole Class** | 0.86  0.97  1  1 |  |
|  | **Interactive Writing** | **Individual**  **Dyads**  **Small Group**  **Whole Class** | 1  1  1  1 |  |
|  | **Checking** | **Roaming**  **Paraphrasing for Clarification**  **Repetition**  **Progress Indication** | 0.67  0.48  0.36  0.99 |  |
|  | **Student Autonomy** | **Assignment**  **Workspace**  **Collaboration**  **Pace** | 1  0.89  0.98  1 |  |
|  | **Added Personnel** | **Assistant**  **Volunteer**  **Guest Writer** | 0.90  0.97  1 |  |
|  | **Homework** |  | 1 |  |
| **Classroom Management** | **Nonverbal Means** | **Light**  **Sound**  **Proximity**  **Gesture**  **Physical**  **Tokens** | 0.96  0.74  0.98  0.89  0.98  0.99 | 0.91 |
|  | **Verbal Means** | **Praise**  **Prompt/Reminder**  **Sanction Model**  **Description** | 0.89  0.33  0.97  0.73 |  |
|  | **Regulation** | **Behavior Planning**  **Goal Setting**  **Progress Monitoring & Evaluation**  **Peer Compliance Request** | 1  1  1  1 |  |
|  | **Punitive** | **Timeouts**  **Posted Warnings**  **Ignoring**  **Response Cost** | 1  1  1  1 |  |
